# Spatial Analysis of Incidence of Diagnosed Type 2 Diabetes Mellitus and Its Association With Obesity and Physical Inactivity

**DOI:** 10.3389/fendo.2021.755575

**Published:** 2021-10-28

**Authors:** Jinrong Wu, Yang Wang, Xin Xiao, Xianwen Shang, Mingguang He, Lei Zhang

**Affiliations:** ^1^ Centre for Eye Research Australia, Royal Victorian Eye and Ear Hospital, Melbourne, VIC, Australia; ^2^ Research Centre for Data Analytics and Cognition, La Trobe University, Melbourne, VIC, Australia; ^3^ Center for Optometry and Visual Science, Department of Optometry, People’s Hospital of Guangxi Zhuang Autonomous Region, Guangxi, China; ^4^ Ophthalmology, Department of Surgery, University of Melbourne, Melbourne, VIC, Australia; ^5^ State Key Laboratory of Ophthalmology, Zhongshan Ophthalmic Center, Sun Yat-sen University, Guangzhou, China; ^6^ Melbourne Sexual Health Centre, Alfred Health, Melbourne, VIC, Australia; ^7^ Central Clinical School, Faculty of Medicine, Monash University, Melbourne, VIC, Australia; ^8^ Department of Epidemiology and Biostatistics, College of Public Health, Zhengzhou University, Zhengzhou, China

**Keywords:** diabetes, incidence, GIS, Australian, risk factors, obesity, physical inactivity

## Abstract

**Objectives:**

To investigate the spatial distribution of 10-year incidence of diagnosed type 2 diabetes mellitus (T2DM) and its association with obesity and physical inactivity at a reginal level breakdown.

**Methods:**

Demographic, behavioral, medical and pharmaceutical and diagnosed T2DM incidence data were collected from a cohort of 232,064 participants who were free of diabetes at enrolment in the 45 and Up Study, conducted in the state of New South Wales (NSW), Australia. We examined the geographical trend and correlation between obesity prevalence, physical inactivity rate and age-and-gender-adjusted cumulative incidence of T2DM, aggregated based on geographical regions.

**Result:**

The T2DM incidence, prevalence of obesity and physical inactivity rate at baseline were 6.32%, 20.24%, and 18.7%, respectively. The spatial variation of T2DM incidence was significant (Moran’s I=0.52; p<0.01), with the lowest incidence of 2.76% in Richmond Valley-Coastal and the highest of 12.27% in Mount Druitt. T2DM incidence was significantly correlated with the prevalence of obesity (Spearman r=0.62, p<0.001), percentage of participants having five sessions of physical activities or less per week (r=0.79, p<0.001) and percentage of participants walked to work (r=-0.44, p<0.001). The geographical variations in obesity prevalence and physical inactivity rate resembled the geographical variation in the incidence of T2DM.

**Conclusion:**

The spatial distribution of T2DM incidence is significantly associated with the geographical prevalence of obesity and physical inactivity rate. Regional campaigns advocating the importance of physical activities in response to the alarming T2DM epidemic should be promoted.

## Introduction

Diabetes is a key non-communicable disease (NCDs) targeted for global action (1, 2). The prevalence of diabetes has increased from 4.7% in 1980 to 8.5% in 2014 in the adult population globally (1). The World Health Organization (WHO) projected that the number of diabetes cases worldwide would reach 366 million by 2030 (3). Type 2 diabetes mellitus (T2DM), diagnosed when the pancreas could no longer produce sufficient insulin, accounts for 90-95% of all diabetic cases (4). The management of T2DM is complex and time-consuming, often involving regular health consultations, lifestyle modifications, frequent blood glucose and podiatry monitoring and complex medication regimes (5–8). The estimated annual spending on T2DM in Australia is around $6 billion. The average annual healthcare cost per person is as high as $4,025, even if there are no associated complications (9).

Previous studies (10–12) have revealed that the development of T2DM results from complex interactions between genetic, environmental, lifestyle and other risk factors. It is also revealed that obesity and physical inactivity are highly correlated with T2DM (13, 14). Most of these associated studies have been conducted on an individual level, providing essential information to identify key modifiable health behaviours for the general health of people living with T2DM. However, understanding the geographical trends of T2DM at a population level may provide important evidence to inform better health policies and population-based prevention programs.

Several existing studies have reported the geographical variance in T2DM disease burden (15–19). Angela et al. (16) evaluated the geographical variations of T2DM incidence among teenagers in the United States. Douglas et al. (17) identified regions with a high disease burden of T2DM in the city of London, UK. Similar studies were also conducted in Ukraine (19) and China (20). Geospatial correlation studies that focused on the association between the risk factors and population effects on T2DM were also conducted in Europe, the US and China as evidence to facilitate political commitment and implementation of community-based programs to curb the epidemic of T2DM (21–23). They reported the association of T2DM with obesity and physical activity, and some studies also highlighted that these geographical variances could result from social determinants, such as income and employment (24–26). In Australia, a typical developed country with the world’s 12^th^ largest economy, several government-issued reports unveiled the geographical variations of T2DM incidence nationwide but without in-depth spatial analysis of T2DM and its interaction with population risk factors (9, 27–29). In particular, more than 90% of T2DM in Australia were found in the middle-aged and elderly population of more than 45 years old. Therefore, understanding the geographical trends and the relevant population factors that contribute to T2DM in this group is key for the prevention of T2DM in the ageing Australian population.

We hypothesised that there are significant geographical variations in T2DM incidence across the Australian state of New South Wales, and these variations are potentially associated with the obesity prevalence and physical inactivity rate in the population. We used demographic, behavioural, medical, and pharmaceutical data from a ten-year follow-up study cohort with 266,896 participants to explore the geographical variation in the incidence of T2DM and associated population factors. Findings from this study will add new evidence to inform health policies to be modified based on geographical variations across Australia at a population level.

## Methods

### Data Source

The data used in this study was mainly collected from the Sax Institute’s 45 and Up Study, a population-based cohort study with participants aged 45 and over in NSW, Australia (30). A total of 266,896 participants, who were first randomly sampled from Services Australia’s (formerly the Australian Government Department of Human Services) Medicare enrolment database, joined the Study between January 2006 and December 2009. Each participant completed a baseline questionnaire and provided signed consent for follow-up and linkage of one’s information to a routine health database. The baseline questionnaire captured a broad range of information related to socioeconomic, health, and lifestyle factors. The 45 and Up Study data was also linked to the Medicare Benefits Schedule (MBS), and Pharmaceutical Benefits Scheme (PBS) claims from 2004 to 2016 with deterministic matching. Hence, detailed medical procedures (identified by MBS code) and medications prescribed by clinicians (identified by PBS code) could be tracked for each participant. Data of population walking to work was obtained from a census study by Zander et al. in 2011 (31), where they aggregated the raw data from the ABS.

### Geographical Measures

Statistical Area Level 3 (SA3) is a regional breakdown of Australia based on the Australian Statistical Geography Standard (ASGS), using a standardised set of numeric codes issued by the ABS to uniformly identify geographical entities (32). The delimitation of a total of 91 SA3s in New South Wales was based on the relative homogeneity in the demographic, functional, geographical, and socioeconomic characteristics (32). SA3 code for each participant was derived from the self-reported baseline questionnaire in the 45 and Up Study, and the SA3 maps of Digital Boundaries for the year 2011 were downloaded from ABS website (32).

### Ethics Considerations

Ethics approval for the 45 and Up Study was granted by the University of New South Wales Human Research Ethics Committee. Ethics approval for this specific study was granted by the Royal Victorian Eye and Ear Hospital Human Research Ethics Committee.

### Inclusion and Exclusion Criteria

We excluded participants with established diabetes at baseline, defined as those who:

• self-reported to have established diabetes;
• applied diabetes medications before the baseline implied by the PBS database (33);
•defined as a diagnosis of diabetes earlier than the last childbirth, but without diabetes medication use subsequently;
•had missing or invalid BMI or Statistical Area 3 data;
•reported age of diabetes diagnosis older than the age at baseline survey. We also excluded participants who are from the SA3 regions with less than 100 participants (10702, 10803 and 12402).

**Figure 1:**
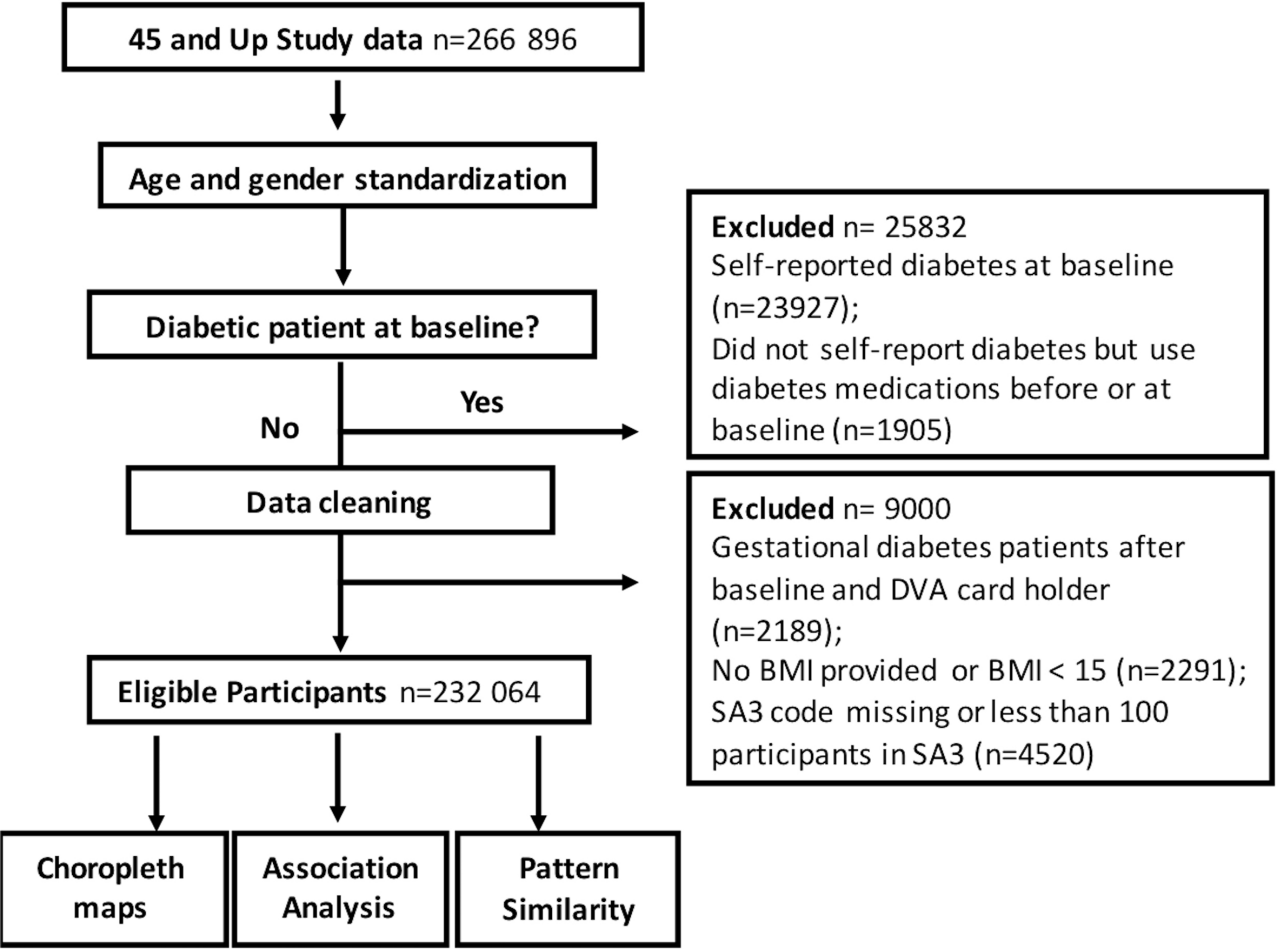
Spatial Analysis of Diabetes Study Flow Diagram.

After exclusions, a total of 232,064 residents were selected in this study (**Figure 1**).

### Outcomes and Associated Factors

The SA3-level 10-year diabetes incidence rates were estimated in 232,064 residents for the period from 2006 to 2017. Age- and-gender adjusted diabetes incidence was calculated using the direct adjustment method, based on the age and gender distribution of 2011 census population data. We defined diabetes incidence as the first occurrence of any kind of medications prescribed for T2DM (including oral hypoglycaemic agents and insulin) with their corresponding PBS codes. As all participants were aged above 45 years, we assumed that all incident cases of new diabetes medication use were for T2DM rather than type 1 diabetes mellitus.

Each participants’ body mass index (BMI) was derived from the self-reported baseline questionnaire in the 45 and Up Study. Definition of obesity is based on body mass index calculated from height and weight, which was previously validated in this study cohort (34, 35). According to the definition by WHO (34), a person with a BMI of more than 30kg/m^2^ is considered obese in our study. Using this standard, we calculated the obesity prevalence for each SA3 region.

We applied two methods to evaluate physical inactivity in our study. Using the first method, we defined people who do physical activities of less than five sessions (metabolically adjusted) per week as participants “having insufficient physical activities”, based on recommendations by the US Centers for Disease Control (CDC) (36, 37). Since information about the number of physical activity sessions for all participants were available in the self-reported baseline questionnaires in the 45 and Up Study, we aggregated the percentage of the population with insufficient physical activities for each SA3 region. To strengthen our physical inactivity measures, we applied a secondary indicator to measure physical inactivity. A previous study (31) conducted in Australia has shown that the percentage of the population walking to work, which is the ratio of the number of journeys to work by walking to the total number of journeys to work, is a good indicator of physical activity in Australia. A low percentage of the population walking to work presented the lack of physical activity in the population and a high rate of physical inactivity. Since the data from the previous study were aggregated based on a different region breakdown standard (38), we mapped each region in that study to the SA3 region in our study using the correspondence data from ABS and re-calculated the percentage of the population walking to work for each SA3 region.

## Analyses for T2DM Incidence

SAS version 9.4 (SAS Institute Incorporation) and R Studio was used for non-spatial data analyses. To understand the distributions in diabetes incidence across different regions in Australia, we calculated the range, mean and standard deviation for each potential association factor, including obesity prevalence, percentage of the population with insufficient physical activities and percentage of the population walking to walk. We further analysed the non-spatial correlation between diabetes incidence and each factor using Spearman correlation.

For spatial analysis, Choropleth maps of the outcome and associated factors were plotted using ArcGIS 10.4 (ESRI, Redlands, CA) and GeoDa 1.12. We applied univariate Moran’ I to identify if there are significant geographical variations in diabetes incidence, obesity prevalence, percentage of the population with insufficient physical activities and percentage of the population walking to walk, respectively. If a univariate Moran’ I value is close to 1, it indicates a significant geographical variation, whereas a value close to 0 indicates no clear geographical variations. To assess colocation between T2DM incidence and each of the association factors, we calculated the bivariate Moran’s I and intraclass correlation coefficient for the associated factors and diabetes incidence. Bivariate Moran’s I was a global measure of spatial correlation to measure the influence one variable has on the occurrence of another variable in close proximity (39). A value close to 1 represents a clear spatial correlation, whereas a value close to 0 means no clear spatial correlation.

## Results

### Characteristics of Participants Based on SA3

Amongst the 232,064 individuals in the 88 SA3s aged 45 and over, the average 10-year incidence of diabetes in the period of 2006–2017 was 6.32% (95% CI, 2.76-12.27%). The average prevalence of obesity across all SA3s was 20.24% (10.36-31.40%). The average percentage of the population with insufficient physical activities was 18.70% (10.39-25.66%), and only 4.43% (1.45 -23.11%) of the population walk to work in metropolitan areas.

### Correlation Between Diabetes and Associated Factors

**Figure 2:**
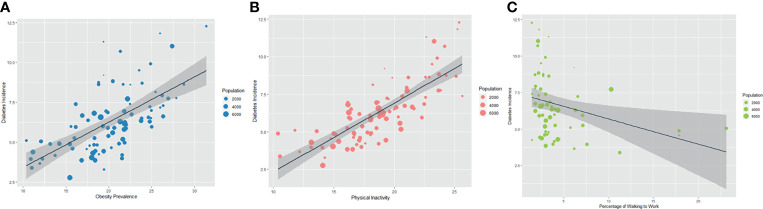
Association in Prevalence of Obesity **(A)** and Physical Inactivity Rate **(B, C)** with Incidence of Diabetes **(A)** shows the correlation between T2DM incidence and obesity prevalence with a spearman correlation coefficient of 0.62 (p < 0.001); **(B)** shows the correlation between T2DM incidence and percentage of the population with insufficient physical activities with a spearman correlation coefficient of 0.79 (p < 0.001); **(C)** shows the correlation between T2DM incidence and percentage of the population walking to work with a spearman correlation coefficient of -0.44 (p < 0.001).

As shown in **Figure 2**, the prevalence of obesity and the percentage of the population with insufficient physical activities showed a strong positive correlation with T2DM incidence (obesity prevalence: *r=*0.62, *p*<0.001, insufficient physical activities: *r=*0.79, *p*<0.001). The percentage of the population walking to work showed a negative correlation with T2DM incidence (*r=*-0.44, *p*<0.001) in metropolitan areas.

### Geographical Variation of Diabetes Incidence and Associated Factors

**Figure 3:**
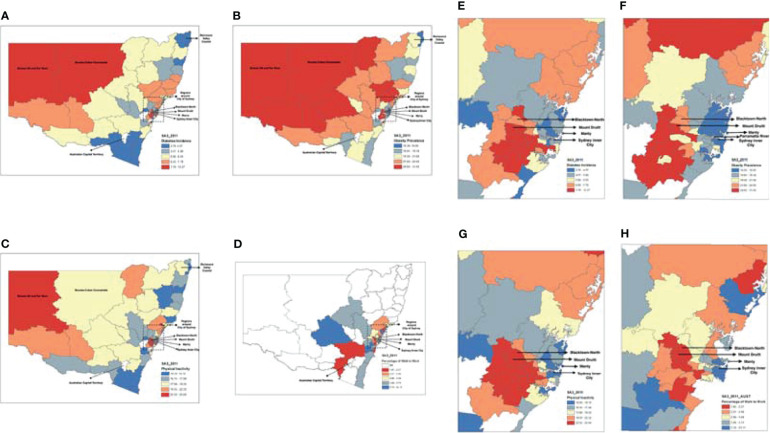
Geographic Variations in the Incidence of Diabetes, Obesity Prevalence, Percentage of the Population with Insufficient Physical Activities and Percentage of the Population Walking to Work. **(A)** shows the variations in T2DM incidence at SA3 level with a Univariate Moran’s I of 0.52 (p=0.001) while **(E)** shows T2DM incidence in the magnified Sydney region; **(B)** shows the variance of obesity prevalence at SA3 level with a Univariate Moran’s I of 0.67 (p=0.001) while **(F)** shows obesity prevalence in the magnified Sydney region, and by comparing between **(A, B)**, it can be identified a very similar pattern between them with a Bivariate Moran’s I of 0.37 (p=0.001) and an intraclass correlation coefficient of 0.6 (p<0.001); **(C)** shows the variance of the percentage of the population with insufficient physical activities at SA3 level with a Univariate Moran’s I of 0.59 (p=0.001) while **(G)** shows the percentage of the population with insufficient physical activities in the magnified Sydney region, by comparing between **(A, C)**, it can be identified a very similar pattern between them with a Bivariate Moran’s I of 0.54 (p=0.001) and an intraclass correlation coefficient of 0.8 (p < 0.001); **(D)** shows the variance of the percentage of the population walking to work at SA3 level with a Univariate Moran’s I of 0.44 (p=0.001) while **(H)** shows the percentage of the population walking to work in the magnified Sydney region, and by comparing between **(E, H)**, it can be identified a similar pattern between them with a Bivariate Moran’s I of -0.23 (p=0.001) and an intraclass correlation coefficient between T2DM incidence and percentage of the population walking to work of -0.47 (p < 0.001); by comparing between, it can be identified a very similar pattern between them with and an intraclass correlation coefficient of 0.61 (p < 0.001).

Geographical variations in T2DM incidence and associated factors were shown in **Figure 3**. T2DM incidence presented a significant uneven geographical distribution (Univariate Moran’s I=0.52; *p*=0.001). Similar findings were also identified in the geographical distribution of prevalence of obesity (Univariate Moran’s I=0.67; *p*=0.001), percentage of the population with insufficient physical activities (Univariate Moran’s I=0.59; *p*=0.001) and percentage of the population walking to work in metropolitan areas (Univariate Moran’s I=0.44; *p*=0.001).

The choropleth maps (**Figure 3**) shows that the north-western regions of NSW and the western regions of Sydney had relatively high T2DM incidence, high obesity prevalence, a high percentage of the population with insufficient physical activities and a low percentage of the population walking to work. In comparison, areas along the coastal lines had relatively low T2DM incidence, low prevalence of obesity, low percentage of the population with insufficient physical activities and a high percentage of the population walking to work.

The highest T2DM incidence was found in Mount Druitt (**Figures 3A, E**), which also showed the highest obesity prevalence (**Figures 3B, F**) and the second-highest percentage of the population with insufficient physical activities (following its neighbour Blacktown-North, **Figures 3C, G**). Consistently, both Blacktown-North and Mount Druitt showed the lowest percentage of the population walking to walk (**Figures 3D, H**). In contrast, the lowest T2DM incidence was found in Richmond Valley-Coastal and Manly (**Figures 3A, E**), which also showed the lowest obesity prevalence. In addition, Sydney Inner City (**Figure 3G**) showed the lowest percentage of the population with insufficient physical activities. In Southeast Sydney, SA3s with high T2DM incidence also presented high obesity prevalence and physical inactivity rate (**Figures 3E**–**G**). Two SA3s, Bourke-Cobar-Coonamble, and Broken Hill, together with far west regions, showed very high T2DM incidence (**Figure 3A**).

### Colocation Between the 10-Years Incidence of T2DM and Associated Factors

Results of the spatial correlation analyses indicate significant colocation between T2DM incidence and its associated factors, such as the prevalence of obesity (Bivariate Moran’s I=0.37, p=0.001), the percentage of the population with insufficient physical activities (Bivariate Moran’s I=0.54, p=0.001) and the percentage of the population walking to work in metropolitan areas (Bivariate Moran’s I=-0.23, p=0.001, **Figure 3**). Similarly, intraclass correlation analysis based on the choropleth maps showed a significant spatial correlation between T2DM incidence and its associated factors (p<0.001 for all, **Figure 3**).

## Discussion

In this large-cohort GIS study of Australians aged 45 and above, we identified significant geographical variations in T2DM incidence across NSW, Australia. We demonstrate that T2DM incidence is significantly correlated with obesity prevalence and physical inactivity rate at a population level.

The geographical variation and spatial clustering of T2DM incidence identified in this study are consistent with the results from previous studies that compare the diabetes prevalence among major cities and remote areas in Australia (40). Besides, the very high T2DM incidence in the outback region of NSW could result from the arid local climate and the lack of health resources in these regions (41).

Our finding that the onset of T2DM was closely associated with obesity and physical inactivity is consistent with the findings from existing studies (13, 42, 43). Although multiple epidemiological studies identified obesity as the most significant associated factor for T2DM due to its pro-inflammatory contribution to the development of insulin resistance and disease progression (44, 45), our study revealed that physical inactivity rate is more important contributing factor to T2DM than obesity in an Australian population. This is supported by Ansari (42) who found that physical activities, including stair climbing and cycling, may reduce the risk of T2DM at a population level.

Previous studies have identified that the spatial variance of T2DM, obesity and physical inactivity can result from a few socioeconomic factors, such as income, education and occupational status (46, 47). It is found that one’s education level is most likely to affect one’s understanding and knowledge of the health benefits of preventative behaviors (48). Low income and education level have also shown to be associated with a high risk of metabolic system, leading to over-weight and obesity (49, 50). In addition, employment status categorized by occupation, has shown an inverse relation with glucose intolerance (47, 51). Therefore, more educational resources and occupational guidance can be provided to population in the high-risk regions for T2DM control.

The major contribution of this study is that we identified a significant colocation of T2DM incidence with obesity prevalence and physical inactivity rate at a population level. A population with a high prevalence of obesity (BMI>30kg/m^2^) and a high percentage of insufficient activity (physical activities of <5 sessions/week) was strongly associated with a high incidence of T2DM. To our knowledge, our study was the first to investigate the spatial correlation of T2DM with its associated lifestyle factors, contributing to the sociocultural perspective of T2DM prevention. The advances in technology and transportation, long hours of sedentary office work, and increasing access to processed food increase the risk of obesity and physical inactivity (52), leading to an increased risk of T2DM onset in adults. For instance, in our study, the Mount Druitt region has the highest obesity prevalence, the second-highest percentage of population physical inactivity; therefore, substantial changes in public policies to create an environment that promotes the wellbeing of the whole community is a priority in the region. Further, the government may consider allocating resources for health promotion campaigns and increasing accessible exercise facilities at the workplace to reduce T2DM incidence (53). Another strength of this study is that the use of a sizable study cohort with 232,064 participants in Australia with a long follow-up period, and the comprehensiveness of the captured information for each participant, can lead to more reliable study findings. T2DM incidence was captured based on the Medicare record system, which effectively minimized the recall bias from the participants, especially the elderly.

However, the study has a number of limitations. First, our definition of T2DM might overlook cases of gestational diabetes. Second, our study was conducted based on the assumption that participants did not move to a new location, and their body weights were relatively stable during the study period. Third, the data of working to work used in this study only relates to the regions around the City of Sydney not the whole of NSW and is limited to the people who are employed. Fourth, data for obesity prevalence and physical inactivity rate were collected from individual-level questionnaires with the response rate of only 18%, raising issues regarding the overall representativeness of the recruited participants from each SA3 region. But we found that the characteristics of participants in our study are, in fact, very similar to the characteristics presented in the New South Wales Population Health Survey conducted by the NSW government (54).

In conclusion, this study is a large-scale GIS study that addressed the geographical disparities in T2DM incidence and its associations with the prevalence of obesity and physical inactivity across an Australian state. This study highlights that a high prevalence of obesity and physical inactivity in a population may contribute to a high incidence of T2DM in the population. Community-based intervention on healthy lifestyles and behaviors should be prioritized to help control T2DM incidence in a population.

## Data Availability Statement

The data analyzed in this study is subject to the following licenses/restrictions: This research was completed using data collected through the 45 and Up Study (www.saxinstitute.org.au) supplied by Services Australia and the Australian Bureau of Statistics (ABS). The 45 and Up Study is managed by the Sax Institute. Requests to access these datasets should be directed to www.saxinstitute.org.au.

## Author Contributions

JW was in charge of the project experiments and analysis, and writing the draft for the paper. YW was responsible for assuring the experiment the results are consistent and modifying the paper. XX was providing method advice and tool advice to make sure appropriate data analysis. XS is responsible for data preprocessing to extract proper data for our project. MH and LZ supervised the project, gave advice on the techniques and direction advice on the project, and helped in paper revision. All authors contributed to the article and approved the submitted version.

## Funding

MH receives support from the University of Melbourne at Research Accelerator Program and the CERA Foundation. The Centre for Eye Research Australia (CERA) receives Operational Infrastructure Support from the Victorian State Government. The specific project is funded by the Australia China Research Accelerator Program at CERA. MH is also supported by the Fundamental Research Funds of the State Key Laboratory in Ophthalmology, National Natural Science Foundation of China (81420108008).LZ is supported by the National Natural Science Foundation of China (Grant number: 81950410639); Outstanding Young Scholars Support Program (Grant number: 3111500001); Xi’an Jiaotong University Basic Research and Profession Grant (Grant number: xtr022019003, xzy032020032); Epidemiology modeling and risk assessment (Grant number: 20200344) and Xi’an Jiaotong University Young Scholar Support Grant (Grant number: YX6J004).

## Conflict of Interest

The authors declare that the research was conducted in the absence of any commercial or financial relationships that could be construed as a potential conflict of interest.

## Publisher’s Note

All claims expressed in this article are solely those of the authors and do not necessarily represent those of their affiliated organizations, or those of the publisher, the editors and the reviewers. Any product that may be evaluated in this article, or claim that may be made by its manufacturer, is not guaranteed or endorsed by the publisher.
